# Great expectations: altricial developmental strategies are associated with more flexible evolution of limb skeleton proportions in birds

**DOI:** 10.1098/rspb.2025.1647

**Published:** 2025-10-08

**Authors:** Andrew Orkney, Priscila S. Rothier, Brandon P. Hedrick

**Affiliations:** ^1^ Department of Biomedical Sciences, Cornell University College of Veterinary Medicine, Ithaca, NY 14853, USA

**Keywords:** evolution, development, ornithology, wing, skeleton

## Abstract

Birds have repeatedly evolved diverse developmental strategies, including multiple origins of sophisticated parental care, making them a model system to explore the consequences of developmental strategy upon phenotypic evolution. Here, we assess evolutionary covariance between limb proportions and ecological diversity of different bird lineages with altricial (high parental care) or precocial (lower parental care) developmental strategies. In addition, we model overall rates of evolutionary divergence between wing and leg skeletal proportions, allowing us to investigate the influence of developmental strategy upon adaptive traits. We show that while wing and leg proportions evolve independently of one another in altricial lineages, conforming to a modular pattern of evolution attested in birds more generally, there are strong correlations between wing and leg trait evolution in precocial lineages. Unlike precocial groups, divergent wing and leg evolution in altricial lineages is associated with access to novel flight-style combinations and is strongly associated with body mass. This suggests an adaptive association with mechanisms of growth and development. Inspection of internal wing proportions within major clades demonstrates that lineages with more altricial developmental strategies explore a wider range of mechanically relevant wing proportions, such as Brachial Index.

## 1. Background

Growth is a great physiological challenge and may impose a trade-off between juvenile survival and adult performance. Different species navigate this compromise under a variety of developmental strategies, from precocial gazelles and dolphins [1,2], typified by advanced early development, to altricial pouch-young in marsupials and metamorphic crustacean larvae [3,4] whose developmental trajectories are less direct. Developmental strategy may determine available adult forms and functions, including locomotion. Developmental strategy is, therefore, likely to be a strong determinant of macroevolution and a key regulator of disparity. Indeed, there is strong evidence that life-history traits such as metamorphosis intersect with the evolution of disparate locomotory styles in insects [5]. Similarly, juvenile traits retained through amphibian metamorphosis may influence their rates of cranial evolution [6]. Unlike insects and amphibians, amniotes—birds, mammals and non-avian reptiles—do not undergo metamorphosis. Thus, when adults and juveniles employ different locomotory styles, the juvenile locomotor organs must be directly repurposed for their adult applications, potentially intensifying the compromise between juvenile and adult functions.

Development also regulates covariance between traits [7–12], causing ‘modular’ patterns of trait evolution, which may influence wider evolutionary dynamics and ecological adaptation [13–17]. Numerous evolutionary transformations in birds have been attributed to changes in trait covariance, such as the evolution of the beak, the origin of flapping flight and the adaptation of the dinosaurian tail into a tail-wing [18,19].

We hence stand to gain a deeper understanding of the mechanisms, and constraints upon, biological evolution by exploring the interface between development, integrated trait evolution and their combined influence on phenotypic evolution. While much work has studied the dichotomous developmental strategies of metatherian|marsupial (suckling their altricial young in pouches) and eutherian (suckling their more precocial young externally) mammals [20–22], their respective developmental strategies are conserved within each clade, frustrating the deconvolution of ancestry and development. Consequently, definitive evidence of developmental constraints upon amniote limb skeleton evolution has remained elusive [23].

In contrast, multiple independent origins of altricial and precocial developmental strategies are inferred across birds [24], ranging from super-precocial species capable of flight and independent feeding almost immediately upon hatching (e.g. Brush-turkeys *Alectura lathami* [25]), to unfeathered super-altricials with underdeveloped limbs and sense organs, and a total dependence on parental care (e.g. sparrows *Passer domesticus* and cuckoos *Cuculus canorus*) [26].

A ‘modular’ pattern of correlated evolution between skeletal proportions has been demonstrated across the avian limb skeleton, whereby wing and leg proportions evolve independently of one another, perhaps representing an important regulator of avian macroevolution [18,27,28]. However, while it is hypothesized the divergent evolution of functionally differentiated structures should facilitate their adaptation to distinct ecological tasks, observed adaptation is characterized by complex relationships that span multiple modules, inviting more complex explanations [27].

Here, we analyse limb bone proportions in precocial and altricial birds that practise many flight styles. After discovering a latent axis of divergent wing and leg evolution, we construct models of birds’ projection upon this axis over their evolutionary history to determine whether developmental strategy is associated with significant differences in rates of divergent evolution between limbs. We compute interspecific flight-style distinctiveness and explore its relationship to wing–leg divergence and test whether wing–leg divergence exhibits a developmentally dependent allometry across birds. Finally, we investigate internal wing proportions and graduated descriptions of development strategy. We predict that both altricial and precocial bird lineages will have distinct wing and leg evolutionary modules, but that evolutionary correlations between modules will be weaker in altricial lineages due to relaxed functional and physiological constraints upon postembryonic growth. Furthermore, we predict that greater wing–leg divergence in altricial birds will facilitate the more rapid adaptation of their wings towards a wider variety of novel flight-style combinations and internal wing proportions.

## 2. Material and methods

### (a) Data

We sourced limb skeletal proportions from the three-dimensional anatomical landmark constellations of 149 species in Bjarnason *et al.* [29] and the aggregate of linear measurements compiled by Brinkworth *et al.* [30]. We extracted bone lengths in mm from the landmark constellations of Bjarnason [29] by finding the Euclidean distances between the termini of the humerus, radius, carpometacarpus, femur, tibiotarsus and tarsometatarsus. We omitted the ulna because its length is synonymous with the radius.

We removed flightless species from the dataset, which possess reduced wing sizes and proportions (e.g. [31]), implying that they do not balance the same developmental constraints and adaptive demands as flying birds. We also omitted species diverging within the last two million years (retaining only one species within each genus), because small errors (e.g. originating from inter-individual differences or measurement precision) at this temporal scale can have a disproportionate influence on model fits. Furthermore, hybridization between closely related species (especially among ducks and geese e.g. [32]) may confound traits through heterosis and distort inferred divergence times. We also removed *Argusianus argus* after a manual review to identify suspicious measurements.

We sourced presence|absence scores documenting flight-style variety across the species in Bjarnason *et al.* [29] from Orkney *et al.* [27], extending upon Taylor [33]. We characterized developmental strategy, adapting the family-level developmental classifications from a subset of five of the graduated system of eight developmental modes of Starck [34]. All species belonging to altricial Grades 1 and 2 were classified as ‘altricial’ and all species belonging to precocial Grades 1–3 as ‘precocial’. Grade 2 altricials exhibit no motor activity upon hatching, lack feathers and have closed eyes, while Grade 1 altricials possess down. Grade 1 precocial birds locomote upon hatching, possess down, pursue parents and procure food, while Grade 2 precocials are guided to food by parents and Grade 3 precocials experience supplementary feeding [34].

When species were duplicated, we deferred to measurements from Bjarnason *et al.* [29]. In total, we investigated 771 species, including 386 altricial and 222 precocial species.

We represented shared ancestry by pruning the avian super-tree of McTavish *et al.* [35], v. 1.4, via the R *clootl* package.

The curated datasets and code required to replicate our results are indexed on Zenodo [36].

### (b) Analyses

#### (i) Evolutionary modules in altricial and precocial birds

Flying bird masses vary by a factor of 10 000, and scale-dependent (allometric) variation in limb bone proportions may obscure subtle patterns of correlated evolution (modularity) that facilitate or impede adaptation. We, therefore, found residuals of limb bone proportions from linear fits of log-transformed limb bone lengths and body masses, under a generalized least-squares regression approach, accounting for self-correlation among species under the assumption that variance across birds has accumulated by a Brownian motion process across phylogeny, following Garland & Ives [37].

To test whether modularity depends on developmental strategy, we decomposed a matrix representing shared ancestry between pairs of bird species and projected the allometric residuals onto its eigenvectors, following Adams & Felice [38]. These transformed residuals are hence no longer self-correlated and can be investigated with parametric approaches. However, absolute sample sizes remain the result of self-correlated evolutionary processes, and we must estimate new effective sample sizes for significance testing.

We explored modular patterns of correlation across the limb skeleton, and how they depend on development, by finding Pearson’s correlations (ρ) between the transformed residuals for all pairwise combinations of bones, in altricial and precocial subsets of bird species.

We can determine if ρ values differ significantly between altricial and precocial sets of birds by finding the area under the tails of the normal distribution, bounded by evaluating Fisher-transformed correlations: $({\text{tanh}}^{-1}(\rho_{1})-{\text{tanh}}^{-1}(\rho_{2}))/\sqrt{\left(1/(n_{1}-3))+(1/(n_{2}-3)\right)}$. Given that our analysis is a regression-type problem, we substituted sample sizes following Bartoszek [39], simplifying his equations under the assumption that the matrix of shared ancestry between birds approximates the self-correlation among species’ traits.

We tested the one-tailed scenario that correlations are weaker in altricial birds (reflecting our hypothesis that altricial birds have experienced a constraint release upon their limb bone evolution, allowing their wing and leg traits to adapt to independent functions more easily).

We also considered multivariate relationships that may exist between wing and leg bone lengths, which could be more complex and may more closely reflect underlying developmental constraints upon bird evolution. In order to quantify divergence from multivariate evolutionary relationships, we must first discover the leading multivariate evolutionary associations between wing and leg traits, and we may define that an orthogonal axis $\vec{\Delta }$ exists to this evolutionary concordance, which describes divergent evolution.

We found the covariance between limb bone lengths across avian phylogeny, assuming a Brownian motion process, following Revell & Harmon [40]. We then isolated the cross-covariance matrix between wing and leg traits, following Escoufier [41], performed a singular value decomposition on this matrix [42,43] and found that the first principal mode/‘salience’ explained almost all wing–leg covariance (see results). This is, therefore, our axis of multivariate ‘concordant’ evolution. We approximated divergence between wing and leg traits ($\vec{\Delta }$) as its orthogonal complement. We projected allometric residuals of individual bird species upon $\vec{\Delta }$ to find estimates of how ‘divergent’ each individual bird’s skeleton is ($\hat{\Delta }$). The magnitude of $\hat{\Delta }$ decreases in species whose wing and leg traits emerge from concordant evolution and increases in species in which wing and leg traits have evolved differently to one another. We hypothesize that altricial species will exhibit a wider dispersion of $\hat{\Delta }$ values than precocial species.

#### (ii) Model fits of divergent evolution between the wing and leg

We tested whether wing–leg divergence $\hat{\Delta }$ depends on developmental (altricial|precocial) strategy. We undertook stochastic character mapping with the *make.simmap*() function of the *phytools* 2.4−4 R package [44] to simulate the evolution of altricial|precocial strategies under an Equal-Rates model, assuming ancestral precociality (e.g. [45,46]). We fitted single-rate (development does not explain wing–leg divergence) and two-rate Brownian motion models (development does explain divergence) of $\hat{\Delta }$ with the *mvBM*() function of the R package mvMORPH 1.1.8 [47]. We conducted a likelihood ratio test with the *LRT*() function to compare model fits.

We constructed visualizations (‘phenograms’) of wing–leg divergence across avian phylogeny under a two-rate model depending on altricial|precocial development, using the *estim*() function of mvMORPH.

We investigated whether $\hat{\Delta }$ intersects with the ecological and phenotypic properties, flight style and body mass, which we might expect to have strong relevance to concordant or divergent adaptations between wing and leg traits. We computed pairwise Euclidean distances of the Gower-transformed flight-style presence|absences in precocial|altricial species from Bjarnason [29], omitting species scored present for <2 flight-style variables (which may reflect that a species’ habits have been poorly documented). We computed a principal coordinate analysis (PCoA) and truncated to only those axes explaining >5% of total variance. Thereafter, we computed the squared-Mahalanobis distances of all birds from the centroid of flight-style variety as $M_{\text{flight}}=D^{2}=(x-\mu {)}^{\prime }\Sigma^{-1}(x-\mu )$, where x are the PCoA scores, μ is the centroid and Σ is the covariance matrix. We hence assume most birds occupy an ecological centre-ground at μ, that flight styles described in Orkney [27] are not strongly biased towards specific parts of avian phylogeny or confounded by birds’ relationships to one another, and that some combinations of flight styles are inherently more likely to emerge than others. We rooted the resulting metric $\sqrt{M_{\text{flight}}}$, to render the values normally distributed, and overlaid it upon our phenogram of $\hat{\Delta }$ evolution. We hypothesized that birds with more divergent wing and leg evolution, that is higher $\left|\hat{\Delta }\right|$, probably have more unusual anatomies and more unusual flight styles: higher $\sqrt{M_{\text{flight}}}$. We, therefore, fitted second-order polynomial models of the form $\sqrt{M_{\text{flight}}}\approx c+\beta_{1}\hat{\Delta }+\beta_{2}{\hat{\Delta }}^{2}+\epsilon$ with the lm() function of base R, for both precocial|altricial subsets of birds.

Furthermore, we speculated that variation in body mass may reveal ontogenetic/growth-related effects upon $\hat{\Delta }$, which may be modified by precocial|altricial developmental strategy. We, therefore, took the body mass estimates pooled from Bjarnason [29] & Brinkworth [30] and fitted linear models of the form ${\text{log}}_{10}(mass)\approx c+\beta \hat{\Delta }+\epsilon$ in precocial|altricial subsets.

#### (iii) Internal wing proportions

Finally, we investigated the relationship between wing–leg divergence, $\hat{\Delta }$ and internal wing proportions. We reviewed how $|\hat{\Delta }-\bar{\Delta }{|}^{\frac{1}{2}}$, a normally distributed variable representing the magnitude of wing–leg divergence in excess of the mean ‘expected’ value $\bar{\Delta }$, varies in an exploratory analysis of internal proportions of the wing skeleton across all the studied birds in our dataset. We computed eigen decompositions of log-transformed internal proportions and took the exponential of values projected on this vector, following Van den Boogaart & Tolosana-Delgado [48], to project principal components upon a ternary space of wing proportions, enabling us to visualize the major modes of differences between species and how they relate to $|\hat{\Delta }-\bar{\Delta }{|}^{\frac{1}{2}}$. We re-colourized this projection in accordance with developmental categorizations in [34], including semiprecocial|semialtricial species and subset the ternary diagram to plot several major clades independently (Cursorimorphae, Phaethoquornithes, Telluraves and Strisores) to explore the generality of latent structure across bird wing proportion evolution.

## 3. Results

### (a) Evolutionary modules depend on developmental strategy

**Figure 1.**
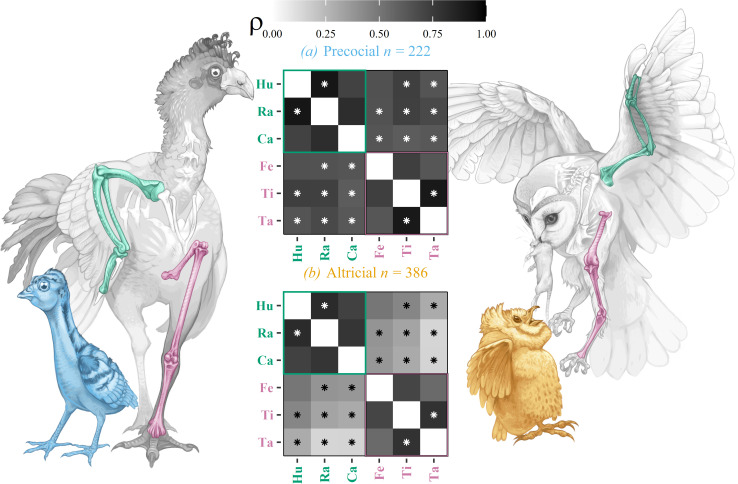
Evolutionary modularity in birds depends on developmental strategy: Pearson's ρ of correlated evolution between pairwise combinations of the allometric residuals of bone proportions, subset by developmental strategy. (*a*)*,(b*) n=386 and n=222 altricial and precocial species. Asterisks indicate p<0.05 results from one-tailed difference of ρ value tests determining if the altricial value is significantly lower, computed on the Fisher-*Z*-transformed ρ values. Correlated evolution between body-mass independent wing and leg proportions is pervasively weaker in altricial birds. Illustrations by Andrew Orkney (2024). hu, humerus; ra, radius; ca, carpometacarpus; fe, femur; ti, tibiotarsus; ta, tarsometatarsus.

Body mass independent wing and leg proportions evolve more independently in altricial lineages (figure 1).

### (b) Divergent evolution between wing and leg is faster in altricial lineages

Divergent evolution between the wing and leg involves divergence in their relative sizes but tends to preserve isometry of internal wing proportions. Vectors of concordant wing and leg evolution coefficients are presented in table 1. The primary axis of covariance between the wing and leg residuals explained >99% of covariance. The vector of wing–leg divergence ($\vec{\Delta }$) is then found as the orthogonal complement to the principal component of concordance, and individual species divergences ($\hat{\Delta }$) are estimated as the projections of allometric residuals upon $\vec{\Delta }$. The sense of coefficients in $\vec{\Delta }$ possess different signs between wing and leg (implied by construction because the transformation matrix from the original to orthogonal complement is a rotation of θ=π/2). The overall pattern described is one of a trade-off between relative wing and leg sizes (recapitulating the evolutionary and ontogenetic coefficients of principal covariation found within six avian families by Watanabe [49]), in which wing variation is roughly isometric, and proximal leg bones have higher weightings than distal leg bones.

**Table 1.** Coefficients of concordant and divergent ($\vec{\Delta }$) wing–leg evolution (three decimal places). Carpo', carpometacarpus; tarso', tarsometatarsus.

	wing			leg		
vector	humerus	radius	carpo'	femur	tibiotarsus	tarso'
concordance	0.582	0.594	0.555	0.539	0.546	0.641
divergence ( $\vec{\Delta }$ )	0.775	0.788	0.844	−0.167	−0.175	−0.087

**Figure 2.**
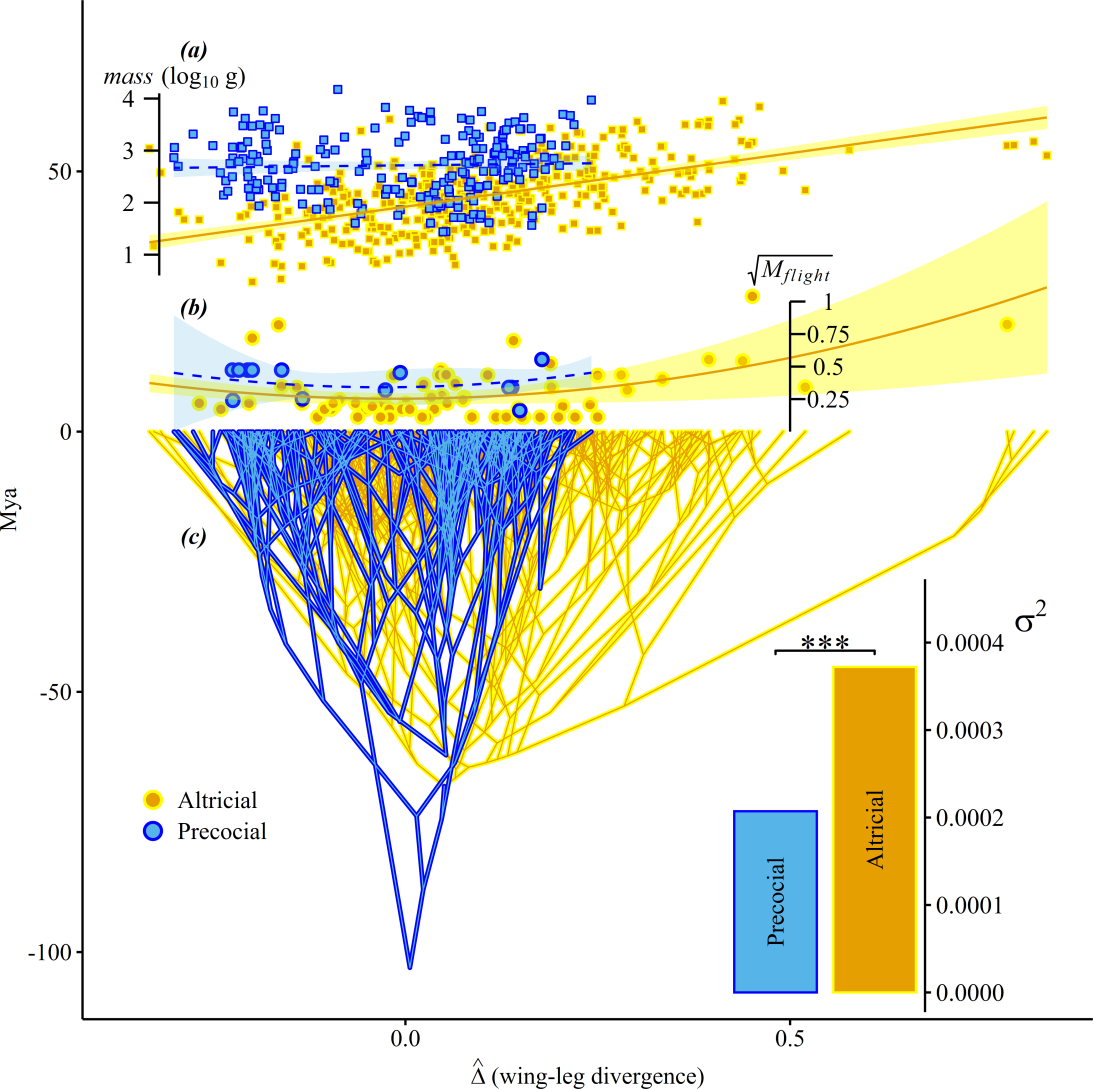
Wing and leg evolution diverges more rapidly in altricial than in precocial lineages. Decoupled wing and leg evolution is associated with the exploration of novel flight styles and coincides with an allometric trend not observed in precocial birds: shallower angles indicate more rapid evolutionary divergence of wing and leg sizes along evolving lineages. (*a*) A linear model of $log_{10}(mass)\approx c+\beta \hat{\Delta }+\epsilon$ finds a significant relationship in altricial (solid orange, n=386) but not precocial (dashed blue, n=222) species. (*b*) Mahalanobis distances of Gower-transformed species-specific flight-style observations ($\sqrt{M_{\text{flight}}}$) are significantly explained by a second-order polynomial $\sqrt{M_{\text{flight}}}\approx c+\beta_{1}\hat{\Delta }+\beta_{2}{\hat{\Delta }}^{2}+\epsilon$ in altricial (solid orange, n=73) but not precocial (dashed blue, n=14) species. (*c*) Phenogram of divergent wing and leg trait evolution ($\hat{\Delta }$, ($log_{10}(mm)$)) across 386 and 222 altricial|precocial species, colourized by estimates of ancestral developmental strategies. The inferred distribution of evolutionary variances ($\sigma^{2}$ ($log_{10}(mm{)}^{2}Mya^{-1}$)) is reconstructed under a two-rate model depending on developmental strategy (corner inset). Mya, millions of years ago. ***: p<0.001.

Variety in $\hat{\Delta }$ across avian phylogeny was better explained by a two-rate Brownian motion model depending on developmental strategy than a single rate model (LRT p<<0.001). Divergent evolution of the wing and leg, $\hat{\Delta }$, is faster in altricial ($3.7\cdot 10^{-4}$$log_{10}(mm{)}^{2}Mya^{-1}$) than precocial lineages ($2.0\cdot 10^{-4}$$log_{10}(mm{)}^{2}Mya^{-1}$) (figure 2) .

### (c) Divergent evolution between the wing and leg is larger in heavier altricial birds

Body size and wing–leg divergence are intimately related in altricial, but not precocial, bird lineages. A linear model (figure 2*a*) shows that body mass variety in altricial species is significantly explained by $\hat{\Delta }$ in the dataset of Brinkworth *et al.* [30] ($p<<0.001,{\;}_{\text{adj}}R^{2}=0.33$), implying a positive allometry of $\hat{\Delta }$ in altricial birds. However, the trend does not differ significantly from 0 in precocial species ($p\approx 0.56,{\;}_{\text{adj}}R^{2}\approx 0$).

### (d) Divergent evolution between wing and leg is associated with novel flight-style variety in altricial birds

Stronger divergence is associated with distinctive flight-style properties in altricial, but not precocial, bird lineages. A second-order polynomial model of $\sqrt{M_{\text{flight}}}$ depending on $\hat{\Delta }$ (see figure 2*b*) reveals a significant quadratic dependency in altricial birds (p<<0.001), while the linear dependency did not differ from zero (p=0.83). Approximately 22% of variance in $\sqrt{M_{\text{flight}}}$ is explained (${\;}_{\text{adj}}R^{2}\approx 0.22)$. No such dependency was found in precocial species (p≈0.55). Species with the lowest $\hat{\Delta }$, indicating disproportionately short wing skeletons, include species of the genera *Archilochus*, *Topaza* and *Chaetura*, whose flight styles are specialist, focused on hovering, hyper-aerial soaring and migration. The closely related *Nyctibius* also has a higher $\sqrt{M_{\text{flight}}}$, on account of its sallying flight, but is distinguished from its relatives by a high $\hat{\Delta }$ value. Species with the highest $\hat{\Delta }$ values, strongly associated with disproportionately long wing skeletons, include species of the genera *Sula*, *Fregata*, *Phalacrocorax* and *Phaethon*, whose flight styles involve pelagic soaring, wing-propelled diving and migration. Curiously, *Pelagadroma* that has a similar flight style to these birds has a very low $\hat{\Delta }$, reflecting its long legs, which it uses to probe the ocean surface for small crustaceans [50]. This behaviour is not represented in our flight-style scores. *Alcedo* and *Chloroceryle*, kingfishers, are distinctive; *Alcedo* employs momentary hovering, while *Chloroceryle* may sally. At least some kingfishers may navigate immersed in water, but this behaviour is not represented in our flight-style scores. Woodland-dwelling birds that fly through clutter typically have $\hat{\Delta }$ values closer to zero (e.g. *Picus*, *Columbina* and *Psittacus*) consistent with concordant wing–leg evolution and lack unconventional flight-style combinations. Precocial species do not typically exhibit either high or especially low $\left|\hat{\Delta }\right|$ (with the exception of *Bonasa*, which is primarily terrestrial, and has a low $\hat{\Delta }$). Nor do precocial species generally exhibit unusual flight styles, but several combine burst and migratory flight (e.g. *Ortalis*, *Odontophorus* and *Mitu*) and exhibit a modest $\sqrt{M_{\text{flight}}}$ increase above background.

### (e) Internal wing proportions show that divergent wing and leg evolution affords altricial birds access to low Brachial Index values

**Figure 3.**
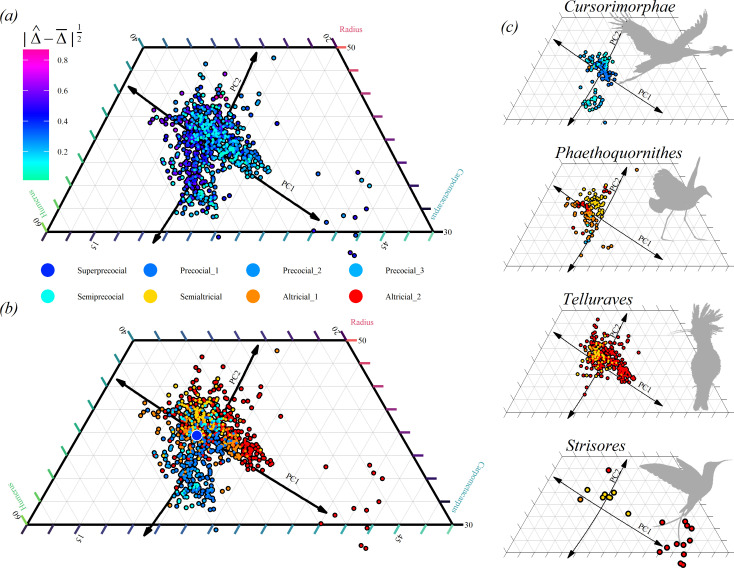
Wing and leg sizes tend to be more divergent in more altricial lineages, which in turn occupy a wider range of potential wing proportions. This pattern is systemic across different bird groups. (*a*) Occupancy of wing proportions across a sample of n=771 extant bird species pooled from Bjarnason *et al.* and Brinkworth *et al.* [29,30], colourized by rooted divergence of the relative wing and leg skeleton proportions ($|\hat{\Delta }-\bar{\Delta }{|}^{\frac{1}{2}}$, units $log_{10}(mm{)}^{\frac{1}{2}}$). Notice that the principal axis of wing proportion evolution (PC1, aligned with variable autopodial contributions) is accessible to birds with lower divergences, but that PC2 (aligned with the ratio of stylopodial to zeugopodial contributions|`Brachial Index') is explored mainly by birds with divergent wing and leg evolution. (*b*) Same, colourized by family-level developmental strategy classifications imputed following Starck [34]. (*c*) Subset plots from top to bottom: Cursorimorphae (gulls and cranes), Phaethoquornithes (tropicbirds, loons, sunbitterns, petrels and their kin, pelecans and their kin), Telluraves (songbirds and their kin, birds of prey, woodpeckers, puffbirds, kingfishers and hornbills) and Strisores (hummingbirds, swifts, tree swiftlets, frogmouths, potoos and the oilbird). Original silhouettes (2024) produced by Isaiah E. Scott, William Coulter Hooker and Andrew Orkney. Silhouettes: *Balearica*, *Pelagodroma*, *Upupa*, *Topaza*. Large dot in (*b*): *Macrocephalon maleo*.

Wing–leg divergence, and its relationship to developmental strategy, is associated with the acquisition of a wider range of wing proportions in altricial birds. A map of $|\hat{\Delta }-\bar{\Delta }{|}^{\frac{1}{2}}$ (the relative magnitude of the differences between wing and leg skeleton lengths, compared with the mean bird) across internal wing skeleton proportions shows low $|\hat{\Delta }-\bar{\Delta }{|}^{\frac{1}{2}}$ values are clustered along the first principal component. This component describes changes in the ratio of the autopod|carpometacarpus lengths (figure 3*a*). Higher $|\hat{\Delta }-\bar{\Delta }{|}^{\frac{1}{2}}$ values are associated with divergence from this axis, along PC2, which describes a trade-off between the humerus|stylopod and radius|zeugopod (Brachial Index = humerus/ulna). Colourizing species by developmental strategy (figure 3*b*) reveals that moderate $|\hat{\Delta }-\bar{\Delta }{|}^{\frac{1}{2}}$ values achieved in more precocial birds are associated with high Brachial Index values (humeralized). However, the very highest ranges of $|\hat{\Delta }-\bar{\Delta }{|}^{\frac{1}{2}}$ are exclusive to more altricial birds, and these are associated with both high and low Brachial Index values, including extremely low values reached in Phaethoquornithes, Strisores and Telluraves (figure 3*c*). Generally, within major avian subclades, species with more precocial developmental strategies are less likely to deviate from PC1 (e.g. semiprecocial species of Cursorimorphae explore both lower and higher PC2 scores than do species of precocial grades 1 and 2, and semialtricial species within Telluraves explore a smaller range of PC2 values than their altricial counterparts.).

## 4. Discussion

Embryogenesis and subsequent growth determine phenotypic variety both within and between species. Changes in the sequence of developmental events [51], the locations of developing structures [52] and the duration over which growth proceeds in distinct parts of the organism [53] can all produce dramatic changes in phenotype. Disentangling the relative influences of embryogenetic and postembryonic controls upon adult phenotypic evolution is hence challenging. Here, we isolate the influence of postembryonic development by exploring variety in the relative proportions of adult wing and leg bones across bird species that differ widely in their rates and styles of postembryonic development between altricial and precocial strategies.

We corroborate our predictions that altricial bird species explore a wider range of internal wing proportions and a greater variety of distinctive flight-style combinations. Precocial birds are typified by early hindlimb-driven locomotion (whether walking or swimming) and tend to undergo a longer, slower period of growth supported primarily by independent feeding [54]. In contrast, altricial birds may not explore the full range of their locomotory capabilities until fledging, and their rapid development is supported exclusively by parental provision. Altricial chicks may experience reduced physiological and mechanical constraints that otherwise exact tolls on developmental potency (e.g. Maynard *et al.* [55]) and limit adult phenotypes.

Our study challenges traditional suggestions that wing–leg divergence emerged in bird-line dinosaurs (in which precocial development has been inferred [56]). We suggest this may explain why the advent of altricial development within crown birds underpinned their post-Cretaceous adaptive diversification, sympathetic with Dyke *et al*.’s suggestion that increased egg sizes facilitated altricial development in Neoaves and relaxed evolutionary constraints [56]. We show that increasingly altricial lineages consistently tend to explore a greater variety of wing proportions, especially Brachial Indices (figure 3*c*). In Strisores, early diverging lineages (nightjars, potoos and the oilbird) are more precocial than later diverging swifts, tree swiftlets and hummingbirds, and occupy a smaller variety of internal wing proportions. Previous authors have suggested low Brachial Index values in particular are integral to the ecological radiation of modern birds [57], although low values are also recorded in some Mesozoic birds presumed precocial [58].

### (a) More altricial development permits divergent evolution of wing and leg proportions

We demonstrate that modular evolution of the bird limb skeleton depends on altricial development (figure 1). In conjunction with recent suggestions of wing–leg integration across the maniraptoran grade [59], this may imply modularity did not emerge as a preadaptation required by flapping flight. We found relatively low divergence between wing and leg skeletal properties in Galloanserae, and higher divergences in many altricial Neoavian clades, contrary to [59]. We suggest that integration between fore- and hindlimb in precocial lineages may arise due to functional demands or physiological and developmental constraints. A greater reliance on wing-assisted incline running in precocial chicks (the deployment of incipient wings to help chicks progress over steep terrain [60]) may require integrated function of the fore- and hindlimb, perhaps limiting opportunities for their phenotypic divergence in adults. Alternatively, precocial species that must expend energy fending for themselves may not be able to support the physiological expense of extended wing growth. Indeed, the final development of wing bone strength and musculature occurs late in postembryonic development (e.g. Carrier *et al.* [61]), perhaps restricting precocial lineages to adult limb proportions that more closely resemble the chick.

### (b) Divergent wing and leg evolution permits access to novel flight styles in altricial birds

Flight-style distinctiveness, $\sqrt{M_{\text{flight}}}$, is significantly related to wing–leg divergence $\hat{\Delta }$ in altricial but not precocial lineages, supporting earlier research showing altricial development affords access to a greater variety of flapping frequencies [62]. Phenograms of $\hat{\Delta }$ (figure 2*c*) show that particularly low or high $\hat{\Delta }$ values are uniquely associated with distinctive combinations of flight styles. We hypothesize the evolution of distinctive flight styles also requires further morphological or physiological changes [63] in addition to novel limb proportions. If altricial development affords birds a greater opportunity to innovate new locomotory styles, it may be possible these lineages also explore a greater diversity of physiologies, which we highlight as a subject for future exploration.

### (c) Mechanistic insight

Figure 2*a* shows that $\hat{\Delta }$ increases with body mass in altricial but not precocial taxa. This may imply growth trajectories leading towards small or large adult sizes are mechanistically relevant to the exploration of unusually high or low $\hat{\Delta }$ values in altricial birds, indicating an ontogenetic basis to variety in $\hat{\Delta }$ across birds. Watanabe previously observed that ontogenetic variation in limb bone lengths within six avian families explained between one- and two-thirds of interspecific variety [49].

If altricial development grants access to disproportionately big wings (and hence the very highest $|\hat{\Delta }-\bar{\Delta }{|}^{\frac{1}{2}}$ values), we suggest that it must critically influence the window of postembryonic growth in which the wing is undergoing elongation. Developmental studies in altricial and semi-altricial species have found that growth rates in the wing continue for an extended period compared with the leg, and that wing development lags behind the leg [61,64,65]. Similar studies of growth rates in *Anas platyrhynchos* demonstrate wing development is delayed in at least some precocial species [64] and may be general among flying birds. Carrier *et al.* [61] hypothesize flight muscle growth, which influences the development of the wing skeleton through plastic interactions [66], is delayed until fledging to minimize the physiological costs incurred sustaining an organ of limited utility to the chick. Building on this idea, we suggest parental food provision to altricial chicks permits greater physiological investment in wing growth during this critical window, permitting extreme wing elongation.

Internal wing proportions in most bird species condense around the principal component describing the relative contribution of the carpometacarpus to the wing length (figure 3*a*). Birds that conform tightly to this axis tend to have relatively low $|\hat{\Delta }-\bar{\Delta }{|}^{\frac{1}{2}}$, and the estimated ancestral condition for avian wing proportions in both altricial and precocial birds falls close to this axis (this can be estimated as $\hat{a}=(1^{\prime }C^{-1}y{)}^{\prime }/\sum C^{-1}$, where C is a matrix of shared ancestry between species pairs and y is a matrix of traits. We remark that the single super-precocial megapode investigated here, *Macrocephalon maleo*—darkest blue point figure 3*b*—possesses proportions synonymous with the ancestral state). The first principal component of wing proportions is aligned with the vector of evolution achieved by ‘random relay’ disruptions to an inhibitory cascade of limb patterning [67]. We suggest lineages’ exploration of PC1 can be mediated during limb bud morphogenesis. In contrast, PC2 is aligned with the ratio of humerus and ulnar lengths—the Brachial Index. Exploration of this axis is related to developmental strategy, and low Brachial Indices are the exclusive domain of altricial lineages (figure 3*b*,*c*). We suggest exploration of PC2 is mediated by postembryonic growth. Our hypothesis is compatible with previous data showing the ratio of carpometacarpus to other wing bones changes strongly before or soon after hatching, while Brachial Indices <1 often emerge in postembryonic growth [68].

### (d) Implications for the evolution of powered flight

Authors including Gatesy, Dial and Middleton [18,69] proposed bipedality in dinosaurs alleviated functional constraints on the forelimb, permitting its independent evolution from the leg and adaptation into a wing. Under this ‘pre-adaptation’ model, we should expect wing–leg divergence to be an integral feature of paravian dinosaur history and to be retained in extant bird lineages in which wings and legs adapt to independent ecological demands. This hypothesis has been partly undermined by the discovery of four winged ‘Tetrapteryx’ dinosaurs [70–73], in which fore- and hindlimb evolution may have been synergistic (as in bats [74]). Pennycuick even hypothesized wing membranes may have contributed to a substantial portion of the flight surface in avian ancestors, and their subsequent loss was permitted only after the evolution of stiff remige aerofoils [75]. Indeed, at least one maniraptoran dinosaur clade is inferred to have possessed extensive wing membranes [76]. Furthermore, it is generally supposed—with the exception of the highly derived super-precocial ratites [77]—that a precocial developmental strategy is ancestral to birds [24]. This hypothesis is supported by low inferred growth rates from basal bird fossils such as *Archaeopteryx lithographica* [78]. This implies the possibility that precocial avian lineages may more closely resemble ancestral birds than do altricial lineages and that, under the pre-adaptation model, precocial lineages should be likely to exhibit divergent evolution between the proportions of their fore- and hindlimb skeletons. The manifest absence of divergent wing and leg evolution in precocial lineages, compounded by its presence in altricial lineages possessing derived developmental and nesting strategies [79], therefore represents a substantial challenge to the pre-adaptation model. Moreover, we note that previous work has suggested the overall limb proportions of basal Avialae were not significantly distinct from other theropods even when confounding factors such as body size and evolutionary relationships are accommodated [80,81]. A further study of fore- and hindlimb proportions in Mesozoic theropods indicated that a similar profile of evolutionary rate shifts across the dinosaur-bird transition occurred in both the Brachial Index and Crural Index (the ratio of tibia and femur lengths) [82]. Together with our neontological work, this suggests the possibility fore- and hindlimb evolution may have been coupled in paravian dinosaurs and only become strongly divergent in altricial lineages of the bird crown group during the Cenozoic. We note with interest that while forelimb proportions in Mesozoic enantiornithid birds occupy a similar distribution to contemporary birds, that enantiornithid hindlimb proportion evolution is extremely conservative [58]. While it is possible that obligate arboreality constrained limb proportion evolution in many enantiornithid birds [83], hindlimb proportions of these birds generally resemble terrestrial palaeognaths and non-avian theropods [80]. Furthermore, enantiornithid fossils of feathered embryos [84] and ground-nesting traces [85] imply a precocial developmental strategy. Enantiornithids also retain wing claws, which might suggest a deeply synergistic evolution between their fore- and hindlimbs extending beyond skeletal proportions to include specification of ectodermal tissues. We suggest that further study is necessary to explore whether wing and leg proportions evolved divergently in enantiornithid birds, and whether precocial development may have limited their exploration of novel limb proportions.

## 5. Conclusion

We demonstrate that altricial developmental strategies in birds may make available more evolutionary routes to new adult forms and novel ecologies. Furthermore, we show that the proportions of the wing and leg are evolutionarily coupled in precocial birds—in stark contrast to the pattern that characterizes avian evolution as a whole. Altricial flying birds explore a wider variety of wing skeletal proportions and divergence between wing and leg proportions than do flying precocial species. Altricial birds are more ecologically diverse, and unusual combinations of flight styles in altricial birds are associated with significantly higher rates of divergent evolution between wing and leg proportions. These results both affirm the anticipated role of developmental strategy in avian locomotory evolution and contribute to an emerging empirical understanding of the balance between multiple controls—developmental, mechanical and adaptive—upon the evolutionary modularity of avian skeletal proportions, reinforcing and refining earlier theoretical work.

## Data Availability

Commented data and codes required to replicate the results in this manuscript are indexed on Zenodo [36]. The repository contains Digital Object Identifiers for publicly available datasets cited in this study. Original datasets from which skeleton measurements were derived can be accessed from [29] and [30]. Original ecological categorizations of avian flight styles can be accessed from [27].
